# Structural, thermochemical and kinetic insights on the pyrolysis of diketene to produce ketene

**DOI:** 10.1007/s00894-023-05572-x

**Published:** 2023-05-03

**Authors:** Pitambar Poudel, Sarah L. Masters

**Affiliations:** School of Physical and Chemical Sciences, University of Canterbury, Private Bag 4800, Christchurch, 8140 New Zealand

**Keywords:** Diketene, Ketene, Computational methods, Pyrolysis, Thermochemistry, Kinetics

## Abstract

Diketene (4-methylideneoxetan-2-one) is a precursor to the formation of either two molecules of ketene, or allene and CO_2_ using pyrolysis techniques. It is not known experimentally which of these pathways is followed, or indeed if both are, during the dissociation process. We use computational methods to show that the formation of ketene has a lower barrier than formation of allene and CO_2_ under standard conditions (by 12 kJ/mol). According to CCSD(T)/CBS, CBS-QB3 and M06-2X/cc-pVTZ calculations the formation of allene and CO_2_ is favoured thermodynamically under standard conditions of temperature and pressure; however, kinetically the formation of ketene is favoured from transition state theory calculations at standard and elevated temperatures.

**Graphical abstract:**

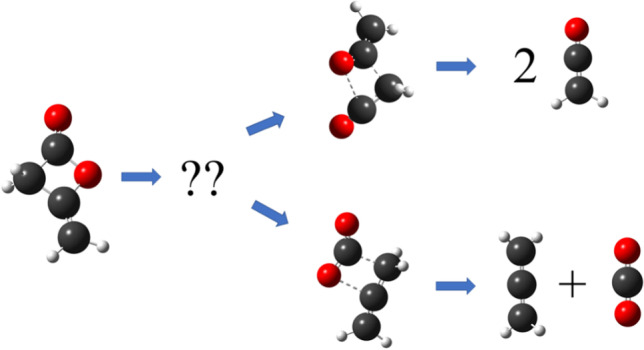

**Supplementary Information:**

The online version contains supplementary material available at 10.1007/s00894-023-05572-x.

## Introduction

Ketenes are a reactive class of organic oxo compounds that are useful in organic synthesis and industrial chemistry [1–5]. For instance, a series of organic compounds were synthesized using ketene as an intermediate in the preparation route [1]. The alkylketene dimer has been used for the preparation of hydrophobic paper sizing agents [2, 4], as well as a hydrophobic starch microcellular foam [5]. Methoxycarbonylketene can be used for the synthesis of the functionalized malonates for agrochemicals and pharmaceuticals [3].

Ketene was first synthesized by Staudinger by reaction of α-chlorodiphenylacetyl chloride with zinc at 452–453 K [6]. Staudinger was seeking to obtain the radical Ph_2_ĊCOCl, inspired by Gomberg who prepared a stable triphenylmethyl radical [7], but the result was the unforeseen discovery of ketene. Ketene has also been prepared by pyrolysis of acetic anhydride using hot platinum wire [8]. This new class of compound dimerized rapidly at room temperature yielding diketene [9].

**Fig. 1:**
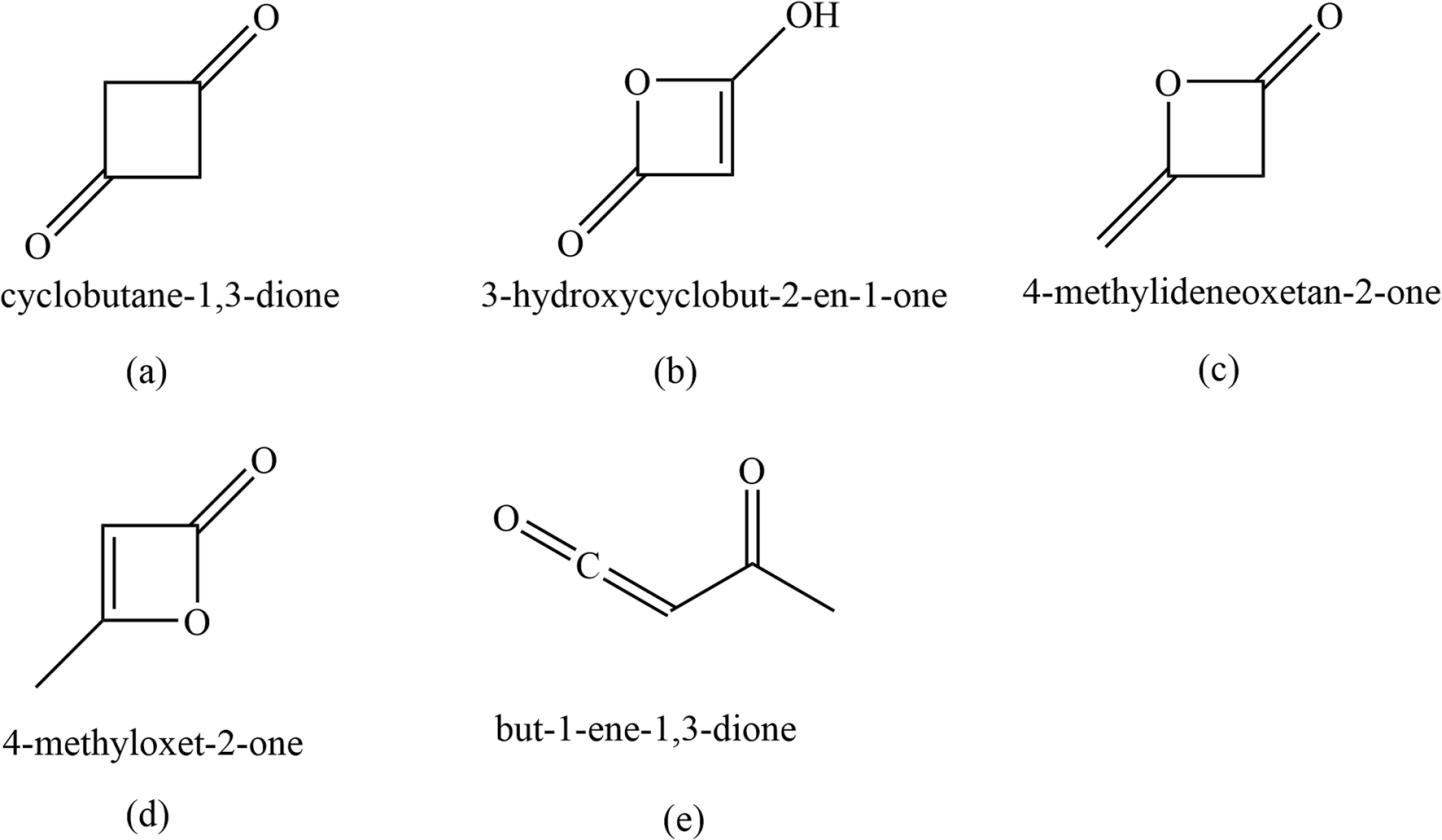
Structure of diketene (**c**) and its other cyclic (**a**, **b** and **d**) and acyclic (**e**) isomers

Diketene is a reactive compound that is useful in synthetic and structural chemistry [10, 11]. Chick and Wilsmore made the first known diketene, as ‘acetylketene’, in 1908 [9]. On standing the liquid or gaseous ketene at room temperature, the new substance [9] was formed as a pungent smelling brown liquid with the possible formula CH_3_COCHCO. Five different isomeric molecular structures were proposed for this compound (one acyclic and four cyclic conformers as shown in Fig. 1) [11].

Boese [6] prepared ketene *via* two different methods, firstly refluxing diketene over hot metal such as platinum or resistant metal and secondly by passing diketene vapour through a hot tube at 823–873 K. The pyrolysis chamber was fitted with a reflux condenser extended to an ice bath to collect the ketene so that unreacted diketene could be observed [11]. The process [11] was about 50% efficient. In 1965 Andreades and Carlson [12] synthesized ketene by pyrolysis of diketene with a flow of nitrogen, which yielded 46–55% ketene. Ketene was prepared from pyrolysis of diluted diketene with ultra-pure argon by thermal decomposition at a temperature of 510–603 K and a constant pressure of 800 Torr [13]. The impurities, such as unpyrolysed diketene and carbon dioxide, were observed in the products by Fourier Transform Infrared spectroscopy (FTIR) investigation, but the quantity was not measured. Previous work from our group [14, 15] generated ketene from pyrolysis of three sources: acetic anhydride, Meldrum’s acid and acetone by using flash vacuum pyrolysis coupled with gas electron diffraction (FVP-GED).

**Fig. 2:**
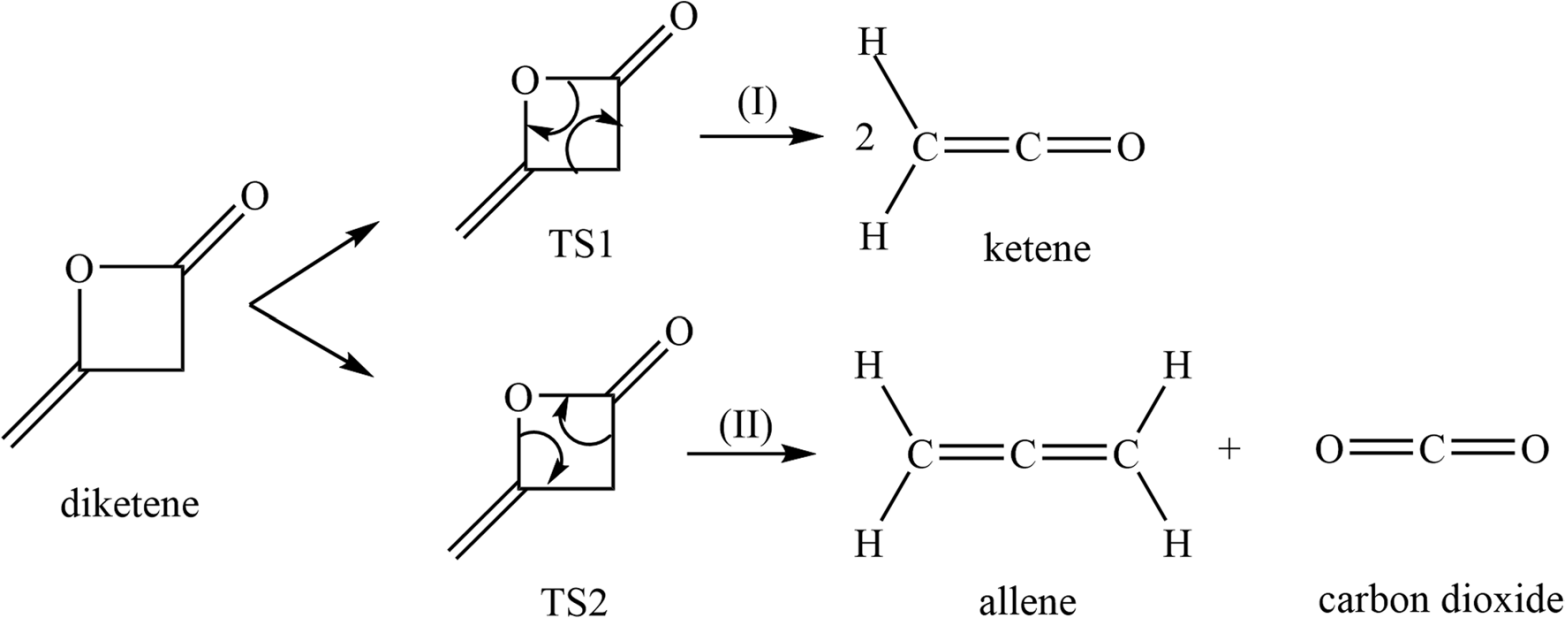
Potential pathways for pyrolytic decomposition of diketene to form (I) ketene and (II) allene and CO_2_

In this work, we use computational methods to study the pyrolysis decomposition of diketene with two possible pathways, (I) and (II). (I) leads to the formation of two equivalent molecules of ketene, and (II) yields allene and CO_2_. Our work provides insight into the underlying mechanism for the pyrolysis decomposition of diketene as shown in Fig. 2 and helps to explain the experimental observations at elevated temperatures.

## Computational details

All calculations were performed using either second order Møller–Plesset perturbation theory (MP2) [16], with the 6-31G*, 6-311G*, 6-311+G*, 6-311++G**basis sets [17–20] and the Minnesota hybrid meta exchange-correlation functional (M06-2X) [21] using the aug-cc-pVTZ basis set [22] with the Gaussian 09 (Revision B.01) [23] and NWChem [24] programs. The results were visualized using GaussView [25]. NWChem [24] calculations were carried out using the resources of the New Zealand e-Science Infrastructure (NeSI). All MP2 methods were frozen core [MP2(FC)]. Geometric optimization of molecules was started at the HF level with a 6-31G* basis set, and frequency calculations were undertaken at MP2/6-311++G** to verify the nature of the stationary points. Thermodynamic parameters and transition state structures with reaction pathways were calculated using the synchronous transit-guided quasi-Newton (STQN) method [26] using CCSD(T)/CBS and composite CBS-QB3 [27] method as described by Curtiss et al. [28, 29]. This method predicts thermochemical parameters with chemical accuracy in the range of mean absolute deviation less than 5.27 kJ/mol [30]. For the CCSD(T)/CBS method, the method is based on extrapolation of the energy to complete basis limit (CBS) using the power function extrapolation scheme suggested by Helgaker et al. [31] (Eq. 1) with the augmented correlation consistent basis sets (aug-cc-pVnZ) of Dunning [32], where *n* = D, T and Q have been used.

1 $$E(X)={E}^{\infty }+\beta\ {X}^{-\alpha }$$

In Eq. 1: *X* is two for double-zeta basis sets, three for triple-zeta basis sets, etc. *E*^∞^ is energy at the basis set limit, and ‘*β*’ and ‘*α*’ are fitting parameters.

The energy profile diagram was plotted from the optimizations at the M06-2X/cc-pVTZ level [21]. The input coordinates were taken from the optimized transition state (TS) structures at the B97D/6-31++G(d,p) level [33]. To ascertain the identity of the relevant transition structures, intrinsic reaction coordinate [34] (IRC) calculations were undertaken at the same level of theory. After IRC calculations followed by structural optimization of the species, it was confirmed that the TS correctly connects the reactant and product(s). Global minima on the potential energy surfaces were identified by the absence of any imaginary vibrational frequencies, and all TS were identified by the presence of one imaginary vibrational frequency.

## Results and discussion

### Quantum chemical calculations

**Fig. 3:**
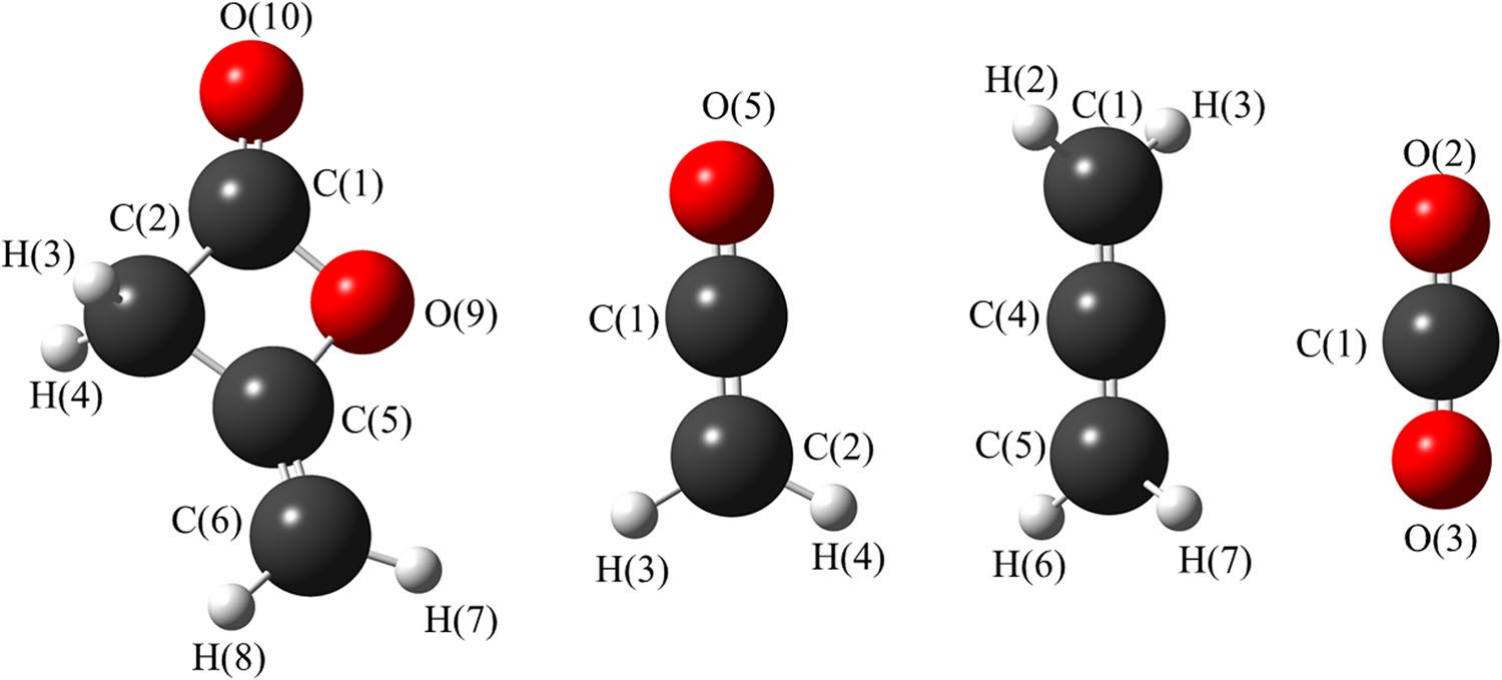
Lowest energy ground state structures (at MP2/6-311++G**) level of (L – R) diketene (*C*_s_), ketene (*C*_2v_), allene (*D*_2d_) and CO_2_ (*D*_∞h_) with atom numbering

Theoretical calculations revealed the ground state structure of diketene has *C*_s_ symmetry and its potential decomposition products ketene, CO_2_ and allene have *C*_2v_, *D*_∞h_ and *D*_2d_ symmetry respectively at both the MP2 and M06-2X levels of theory with different basis sets. The structural parameters for each molecule for the various calculations are given in the supporting information (SI) Table S1. Calculated coordinates for each structure in Table S1 are given in the SI, Tables S2–S6. The structures with atom numbering are shown in Fig. 3.

The gas-phase and solid-state structure of diketene was previously investigated using gas electron diffraction [35] and single crystal X-ray diffraction [36, 37] as well as ^1^H NMR spectroscopy [38] and IR spectroscopy [39, 40]. The previous GED structure [35] differs from the computed structure at MP2/6-311++G** level for various parameters such as *r*C=C, *r*C–C and *r*C–C (for ∠C–C=O) by 2.0 pm, 1.4 pm and 0.9 pm respectively. The bond angles differ by a range of 0.5 to 6.5°, particularly for ∠O–C=O, ∠C–C=O and ∠C=C–C (6.5°, 5.6° and 3.8° respectively). The most recent single crystal X-ray diffraction structure of diketene [37] is in very good agreement with the computed structure. We compared the computed structures of diketene and ketene using MP2 level of theory with previous experimental and theoretical results as shown in Tables 1 and 2.

**Table 1:** Structural parameters for diketene from ab initio, single crystal X-ray diffraction and GED studies^a, b^

Parameters	MP2/ 6-311++G**	X-ray-1 (*C*_1_) [41]	X-ray-2 (*C*_1_) [36]	X-ray-3 (*C*_1_) [37]	GED (*C*_s_) [35]	CCSD(T)/ ANO1(*C*_s_) [37]
*r*C=O	118.9	124.0(6)	122.0(3)	119.1(9)	119.0(4)	118.7
*r*C=C	133.0	135.0(6)	132.0(3)	131.5(11)	131.0(4)	132.5
*r*C–O methoxy	141.1	139.0(6)	147.0(3)	142.9(9)	141.0(4)	139.7
*r*C–O carboxy	140.1	140.0(6)	139.0(3)	138.5(9)	141.0(4)	139.7
*r*C–C_ring_ adjacent to C=CH_2_	150.7	148.0(6)	154.0(3)	150.3(10)	152.0(4)	150.9
*r*C–C_ring_ adjacent to C=O	152.9	146.0(6)	151.0(3)	151.6(11)	152.0(4)	153.0
∠C=C–O	126.5	130.0(20)	126.9(15)	126.1(7)	130.0(4)	126.6
∠C=C–C	139.8	136.0(20)	141.6(15)	141.3(7)	136.0(4)	140.1
∠O–C(C)–C	93.6	94.0(20)	91.3(15)	92.6(5)	95.0(4)	93.3
∠C–O–C	90.8	89.0(20)	90.0(15)	90.6(5)	89.0(4)	91.1
∠O–C(O)–C	93.1	94.5(20)	95.8(15)	93.8(5)	95.0(4)	93.0
∠C–C–C	82.5	83.0(20)	83.0(15)	83.0(5)	81.0(4)	82.6
∠O–C=O	127.5	121.0(20)	123.1(15)	126.1(7)	121.0(4)	127.6
∠C–C=O	139.4	145.0(20)	140.9(15)	140.1(7)	145.0(4)	139.3

^a^All bond distances (*r*) in pm and bond angles (∠) are in degrees (°)

^b^Figures in parentheses are the estimated standard uncertainties (standard deviation) of the last digits at the limits of error

**Table 2:** Structural parameters for ketene from ab initio, single crystal X-ray diffraction and GED studies^a, b^

Parameters	MP2/6-311++G**	GED-1 [42]	MW [43]	GED-2 [14]
*r*C=C	132.2	130.0(20)	131.5(3)^d^	131.3(11)
*r*C=O	116.8	116.0(20)	116.0(10)^d^	114.8(10)
*r*C–H	108.0	107.0(20)^c^	107.5(1)^d^	108.4(7)
∠H–C–H	121.8	117.5(125)^c^	122.0(25)^d^	122.1(10)

^a^All bond distances (*r*) in pm and bond angles (∠) are in degrees (°)

^b^Figures in parentheses are the estimated standard deviation of the last digits

^c^Assumed parameters

^d^Corrections obtained from the harmonic force constants (calculated on the assumption of a simple harmonic oscillator)

Calculated parameters from MP2 level of theory for ketene are in good agreement with the experimental structures from microwave spectroscopy and GED. There were some small differences in the bond distances and bond angles between our theoretical calculation at MP2/6-311++G** and the previous GED-2 study [14], such as the bond distances of *r*C=O (2.0 pm), *r*C–H (0.4 pm) and ∠H–C–H (0.7°).

### Thermochemical calculations

Thermochemical properties such as the Gibbs energy (Δ*G°*), enthalpy (Δ*H°*) and entropy (Δ*S°*) changes were calculated at the CBS-QB3 and CCSD(T)/CBS level of theory (Table 3). The calculated energies and thermochemical parameters are given in SI (Table S7).

**Table 3:** Calculated thermochemical parameters of reaction pathways (I) and (II) calculated from CCSD(T)/CBS (Energy; Eq. 1) with MP2/cc-pVTZ (*H*corr. and *G*corr.) and CBS-QB3 levels of theory for decomposition of diketene at 298.15 K^a^

Reaction pathway	(I)		(II)	
Thermochemical parameters	CCSD(T)/CBS	CBS-QB3	CCSD(T)/CBS	CBS-QB3
∆*H°*	92.6	78.1	−21.5	−23.0
∆*G°*	38.7	25.1	−68.8	−68.3
∆*S°*	181.0	178.0	158.5	151.9

^a^All energies are in kJ/mol except ∆*S°* which is in J/mol/K

The decomposition of diketene to form allene and CO_2_ was found to be exothermic and spontaneous under standard thermodynamic conditions of temperature and pressure. We expected the calculated formation of ketene to be spontaneous; however, the calculations indicated that it was not under standard conditions. Given this unexpected result, thermochemical parameters were also predicted at elevated temperatures. The calculations gave different thermochemical correction parameters, such as *H*corr and *G*corr, which were then used to obtain the thermochemistry of the two reaction pathways for the decomposition of diketene at that temperature. The same energies from the CCSD(T)/CBS calculations, as shown in the SI (Table S7), were used. It was found that the change in temperature affects the thermochemical parameters of both reactions quite dramatically as shown in Table 4. The calculations were performed at three different temperatures, the lowest possible decomposition temperature was taken as 653 K and the highest possible temperature was assumed to be 823 K, along with standard room temperature 298.15 K for comparison. From this it was observed that the formation of ketene was favoured at elevated temperatures with −Δ*G°*.

**Table 4:** Predicted thermochemical properties at different temperatures (298.15 K, 653 K and 823 K) for pathways I and II taken from CCSD(T)/CBS (Energy; Eq. 1) extrapolation with MP2/cc-pVTZ (*H*corr and *G*corr)^a^

	(I)			(II)		
Properties	298.15 K	653 K	823 K	298.15 K	653 K	823 K
∆*H*	92.6	94.5	93.3	−21.5	−21.4	−22.8
∆*G*	38.6	−27.3	−58.8	−68.8	−125.6	−152.5
∆*S*	181.0	186.5	184.9	158.5	158.4	157.6

^a^All the values are in kJ/mol except ∆*S*, whose values are in J/mol/K

### Kinetic calculations

**Fig. 4:**
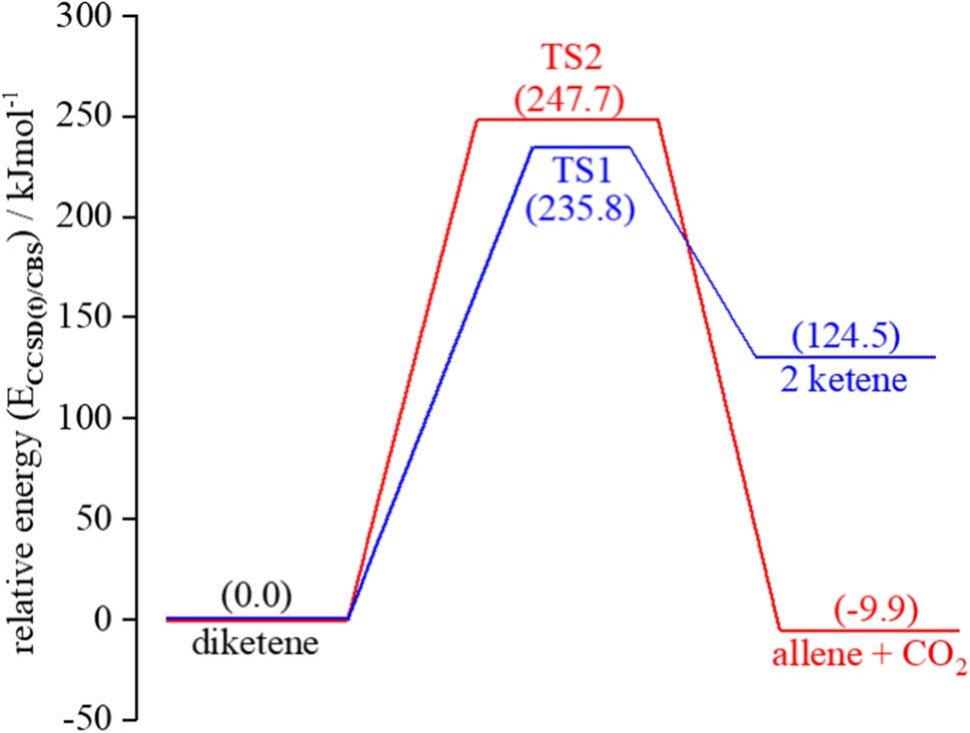
Energy profile diagram for pathways I and II (TS1 and TS2 respectively) for decomposition of diketene at 298.15 K using CCSD(T)/CBS level of theory. Relative energies are given in kJ/mol

**Fig. 5:**
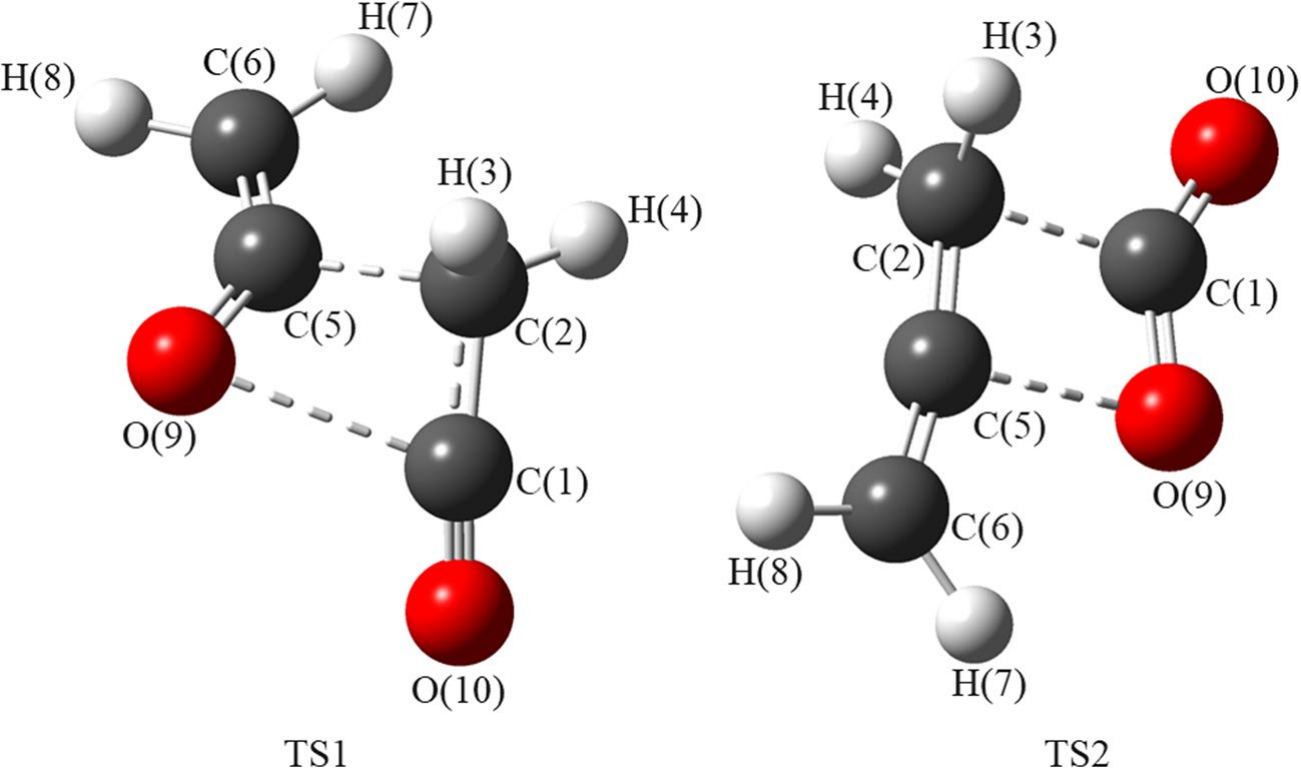
Predicted TS structures, optimized at the M06-2X/cc-pVTZ level with atom numbering. TS1 leads to 2 equivalents of ketene and TS2 leads to allene and CO_2_

The proposed pyrolysis decomposition pathways of diketene were plotted as shown in Fig. 4 with the corresponding TS structures (TS1 and TS2) shown in Fig. 5. Of the two pathways, the formation of ketene (pathway I) was favoured kinetically (*E*_a_ ~236 kJ/mol) compared to that for the formation of allene and CO_2_ (pathway II; *E*_a_ ~248 kJ/mol) at standard temperature (298.15 K). Relative energies (at the CCSD(T)/CBS level of theory) of all species in the pathway reactions are given in the SI (Tables S7 and S8). Calculated coordinates of TS structures are given in SI (Tables S9 and S10).

A previous experimental kinetic study [44] found the relative activation energy of ketene formation by pyrolysis of diketene was 209.2 kJ/mol. The reverse dimerization process was found to have an activation energy of 129.7 kJ/mol by extrapolating the Arrhenius plot from the experimental reaction flow system. In our work, the calculations at CBS-QB3 and CCSD(T)/CBS level return energies that are 235.8 kJ/mol and 98.8 kJ/mol & 193.7 kJ/mol and 111.3 kJ/mol respectively for the decomposition and dimerization of ketene at 298.15 K.

Previous computational work predicted that pathway I is favoured kinetically using both G2M and BAC-G3B3 levels of theory (*E*_a_ for pathway I ~190.0 kJ/mol and pathway II ~200.0 kJ/mol) [13]. As discussed earlier, we have predicted the thermochemical parameters by using a computationally demanding and accurate level of theory, CCSD(T)/CBS, which has not been done before. This finding suggested that the decomposition of diketene still favours pathway II to form allene and CO_2_ at elevated temperatures, but the formation of ketene also becomes energetically more favourable. The kinetic calculations showed that the small difference in *E*_a_ facilitates the formation of ketene at all temperatures, although we predict that allene and CO_2_ should still be formed in observable quantities. This observation explains the early experimental observations [13, 44] of significant levels of allene in the product stream.

Thermal equilibrium constants and transition state theory (TST) rate coefficients at different temperatures were calculated using the modular program Kinetic and Statistical Thermodynamic Package (KiSThelP) version 2021 [45] in Java runtime environment using the Gaussian output files of reactant, TSs and products (M06-2X/cc-pVTZ level of theory) for reactions (I) and (II). The calculated values are given in the SI (Table S11). The ratios of equilibrium constants of reaction (I) to reaction (II) (taken from output file of the program KiSThelP [45]) were 9.36×10^−17^, 1.50×10^−7^ and 6.12×10^−6^ respectively at 298.15, 653 and 823 K. Both reactions (I) and (II) are predicted to occur spontaneously at 653 and 823 K with Δ*G* < 0, Δ*S* > 0 and *K* > *1*. By applying transition state theory, KiSThelP [45] was used to determine the rate constants at 298.15 K. These values for reaction (I) and (II) were calculated as 2.34×10^−21^ and 4.81×10^−23^ s^−1^. Their branching ratios were 3.44 at 653 K and 2.25 at 823 K.

### Mechanism of decomposition

Figure 2 (above) shows two possible pathways for the decomposition of diketene, both following a concerted single-step mechanism. The concerted nature of both TS1 and TS2 were confirmed by the internal reaction coordinate calculations. It would be expected that, due to the high electron affinity of the oxygen atom in the diketene ring, formation of ketene would be favoured via a (2+2) retro-Diels-Alder mechanism [46, 47]; however, this is not what we observed from our calculations or indeed what was observed experimentally. The transition states are very close in energy meaning that both pathways are likely to accessed leading to formation of allene and CO_2_ as well as ketene. Ketene is known to dimerize rapidly to diketene helping explain why it is difficult to observe experimentally. The process is kinetically (*E*_a_ = 111.3 kJ/mol at M06-2X/cc-pVTZ) and thermodynamically (Δ*G* = −38.6 kJ/mol at CCSD(T)/CBS) favourable at room temperature, which was not explained in the previous study [14].

## Conclusions

Our work reveals that the thermal decomposition of diketene occurs *via* a single-step concerted mechanism, supported by the prediction of the transition state structures, TS1 and TS2. Kinetically, pathway I to form ketene (*E*_a =_ 235.8 kJ/mol at M06-2X/cc-pVTZ) is favoured. The formation of allene and CO_2_ is thermodynamically feasible with Δ*G* = −21.5, −23.0 and −59.6 kJ/mol respectively at CCSD(T)/CBS, CBS-QB3 and M06-2X/cc-pVTZ level of theory under standard conditions of temperature and pressure (298.15 K and 1.0 atm). At elevated temperatures (653 and 823 K) both reactions satisfy the condition of spontaneity (Δ*G* < 0, Δ*S* > 0 and *K* > *1*). Transition state theory was applied to calculate the ratios of rate constants for reaction (I) to reaction (II) as 48.60, 3.44 and 2.25 respectively at 298.15, 653 and 823 K. It was observed that the formation of ketene dominates at all calculated temperatures and the presence of allene and CO_2_ can be explained from the thermodynamic analysis.

## Supplementary information

ESM 1: Optimized structural parameters for all molecules involved in reactions (I) and (II). Calculated Cartesian coordinates at different levels of theory for all molecules including transition states. Energies and corrections for thermochemical and kinetic calculations. Calculated equilibrium constants and rate constants and their ratios for diketene decomposition reactions (I) and (II) at different temperatures.

## Data Availability

Available on request.
