# Supplementary material for: Structural, thermochemical and kinetic insights on the pyrolysis of diketene to produce ketene
Source: J Mol Model. 2023 May 3;29(5):168. doi: 10.1007/s00894-023-05572-x (PMC10156866; doi:10.1007/s00894-023-05572-x)
Supplement: Supplementary file 1 — Optimized structural parameters for all molecules involved in reactions (I) and (II). Calculated Cartesian coordinates at different levels of theory for all molecules including transition states. Energies and corrections for thermochemical and kinetic calculations. Calculated equilibrium constants and rate constants and their ratios for diketene decomposition reactions (I) and (II) at different temperatures. [file 894_2023_5572_MOESM1_ESM.docx]

**Supporting Information**

**Structural, Thermochemical and Kinetic Insights on the Pyrolysis of Diketene to Produce Ketene**

Pitambar Poudel and Sarah L. Masters

School of Physical and Chemical Sciences, University of Canterbury, Private Bag 4800, Christchurch, 8140, New Zealand

**Supporting Information**

Optimized structural parameters for all molecules involved in reactions (I) and (II).

Calculated Cartesian coordinates at different levels of theory for all molecules including transition states.

Energies and corrections for thermochemical and kinetic calculations.

Calculated equilibrium constants, reaction quotients and rate constants and their ratios for diketene decomposition reactions (I) and (II) at different temperatures.

Table S1: Optimized structural parameters for diketene, ketene, carbon dioxide and allene at different levels of theory and various basis sets.^a^

| Parameters | MP2/  6-31+G* | MP2/  6-311G* | MP2/  6-311+G* | MP2/  6-311++G** | M06-2X/  aug-cc-pVTZ | |
| --- | --- | --- | --- | --- | --- | --- |
| Diketene |  |  |  |  |  | |
| *r*C(1)=O(10) | 120.1 | 118.8 | 119.0 | 118.9 | 117.8 | |
| *r*C(5)=C(6) | 132.9 | 132.8 | 132.9 | 133.0 | 131.3 | |
| *r*C(1)-O(9) | 140.6 | 140.0 | 140.0 | 140.0 | 138.3 | |
| *r*C(5)-O(9) | 142.3 | 140.1 | 141.2 | 141.1 | 140.2 | |
| *r*C(1)-C(2) | 152.5 | 152.9 | 152.8 | 152.9 | 152.3 | |
| *r*C(2)-C(5) | 150.5 | 150.6 | 150.6 | 150.1 | 150.4 | |
| *r*C(5)-C(6) | 132.9 | 132.8 | 132.9 | 132.9 | 131.3 | |
| *r*C(2)-H(3/4) | 109.3 | 108.2 | 109.2 | 109.1 | 108.7 | |
| *r*C(6)-H(7) | 108.4 | 108.3 | 108.4 | 108.3 | 107.9 | |
| *r*C(6)-H(8) | 108.3 | 108.2 | 108.3 | 108.2 | 107.8 | |
| ∠C(5)=C(6)-H(8) | 119.9 | 120.0 | 119.9 | 119.5 | 119.7 | |
| ∠C(5)=C(6)-H(7) | 121.0 | 120.7 | 121.0 | 120.8 | 120.8 | |
| ∠O(9)-C(5)=C(6) | 126.1 | 126.7 | 126.4 | 126.5 | 126.9 | |
| ∠O(9)-C(1)=O(10) | 127.6 | 127.5 | 127.5 | 127.5 | 127.6 | |
| ∠C(1)-O(9)-C(5) | 90.5 | 90.7 | 90.8 | 90.8 | 91.4 | |
| ∠O(10)=C(1)-C(2) | 139.3 | 139.4 | 139.4 | 139.4 | 139.4 | |
| ∠C(1/5)-C(2)-H(3/4) | 114.1 | 114.2 | 114.1 | 114.0 | 114.2 | |
| ϕH(3)-C(2)-C(1)=O(10) | 64.0 | 64.3 | 64.2 | 64.4 | 64.5 | |
| Ketene |  |  |  |  |  | |
| *r*C(1)=O(5) | 118.2 | 116.9 | 116.9 | 116.8 | 115.5 | |
| *r*C(1)=C(2) | 132.3 | 132.0 | 132.1 | 132.2 | 130.7 | |
| *r*C(2)-H(3/4) | 108.1 | 108.0 | 108.0 | 108.0 | 107.7 | |
| ∠H-C=C(11) | 119.6 | 119.5 | 119.5 | 119.1 | 119.1 | |
| CO_2_ |  |  |  |  |  | |
| *r*C(1)=O(2/3) | 118.1 | 116.9 | 117.0 | 117.0 | 115.5 | |
| Allene |  |  |  |  |  | |
| *r*C(4)=C(1/5) | 131.6 | 131.3 | 131.4 | 131.4 | 129.9 | |
| *r*C(1/5)-H | 108.6 | 108.6 | 108.6 | 108.6 | 108.2 | |
| ∠H-C(1/5)=C(4) | 121.2 | 121.1 | 121.1 | 120.9 | 120.9 |  |

^a^ All bond distances (*r*) in pm, and bond angles (∠) and dihedral angles (ϕ) are in degree (°)

Table S2. Calculated coordinates at the MP2/6-31+G* level for diketene, ketene, allene and CO_2._

| Atom | *x* | *y* | *z* |
| --- | --- | --- | --- |
| Diketene |  |  |  |
| C | 0.05586 | 1.15104 | 0.00002 |
| C | 1.05035 | -0.00506 | 0.00000 |
| H | 0.09712 | 1.77484 | 0.89701 |
| H | 0.09710 | 1.77487 | -0.89694 |
| C | -0.95805 | 0.03892 | 0.00002 |
| C | -2.27332 | -0.15114 | -0.00003 |
| H | -2.94208 | 0.70105 | -0.00005 |
| H | -2.69351 | -1.15016 | -0.00004 |
| O | 0.03597 | -0.97915 | 0.00004 |
| O | 2.23807 | -0.18375 | -0.00004 |
| Ketene |  |  |  |
| C | 0.00000 | 0.00000 | 0.09967 |
| C | 0.00000 | 0.00000 | 1.28145 |
| H | 0.00000 | 0.00000 | -1.22284 |
| H | 0.00000 | 0.94061 | -1.75631 |
| O | 0.00000 | -0.94061 | -1.75631 |
| Allene |  |  |  |
| C | -1.31579 | 0.00000 | 0.00000 |
| C | 1.31579 | 0.00000 | 0.00000 |
| H | 1.87868 | 0.92902 | 0.00150 |
| H | -1.87868 | 0.00150 | -0.92901 |
| H | -1.87868 | -0.00150 | 0.92902 |
| C | 0.00000 | 0.00000 | 0.00000 |
| H | 1.87868 | -0.92902 | -0.00150 |
| CO_2_ |  |  |  |
| C | 0.00000 | 0.00000 | 0.00000 |
| O | 0.00000 | 0.00000 | 1.18087 |
| O | 0.00000 | 0.00000 | -1.18087 |

Table S3. Calculated coordinates at the MP2/6-311G* level for diketene, ketene, allene and CO_2_

| Atom | *x* | *y* | *z* |
| --- | --- | --- | --- |
| diketene |  |  |  |
| C | 0.05305 | 1.15423 | 0.00000 |
| C | 1.04961 | -0.00489 | 0.00000 |
| H | 0.08911 | 1.77555 | 0.89665 |
| H | 0.08911 | 1.77555 | -0.89665 |
| C | -0.94896 | 0.02990 | 0.00000 |
| C | -2.26492 | -0.15212 | -0.00000 |
| H | -2.92871 | 0.70232 | -0.00000 |
| H | -2.68522 | -1.15000 | -0.00000 |
| O | 0.03851 | -0.97388 | 0.00000 |
| O | 2.22437 | -0.18439 | -0.00000 |
| Ketene |  |  |  |
| C | 0.00000 | 0.00000 | 0.10338 |
| C | 0.00000 | 0.00000 | 1.27214 |
| H | 0.00000 | 0.00000 | -1.21676 |
| H | 0.00000 | 0.94003 | -1.74842 |
| O | 0.00000 | -0.94003 | -1.74842 |
| Allene |  |  |  |
| C | 0.00000 | 0.00000 | -1.31312 |
| C | 0.00000 | 0.00000 | 1.31312 |
| H | 0.00000 | 0.92932 | 1.87410 |
| H | 0.92932 | 0.00000 | -1.87410 |
| H | -0.92932 | 0.00000 | -1.87410 |
| C | 0.00000 | 0.00000 | 0.00000 |
| H | 0.00000 | -0.92932 | 1.87410 |
| CO2 |  |  |  |
| C | 0.00000 | 0.00000 | 0.00000 |
| O | 0.00000 | 0.00000 | 1.16906 |
| O | 0.00000 | 0.00000 | -1.16906 |

Table S4. Calculated coordinates at the MP2/6-311+G* level for diketene, ketene, allene and CO_2_

| Atom | *x* | *y* | *z* |
| --- | --- | --- | --- |
| diketene |  |  |  |
| C | 0.05506 | 1.15461 | 0.00000 |
| C | 1.05008 | -0.00512 | 0.00000 |
| H | 0.09337 | 1.77558 | 0.89696 |
| H | 0.09337 | 1.77559 | -0.89696 |
| C | -0.95148 | 0.03376 | 0.00000 |
| C | -2.26746 | -0.15358 | 0.00000 |
| H | -2.93254 | 0.70035 | 0.00000 |
| H | -2.68772 | -1.1519 | 0.00000 |
| O | 0.03848 | -0.97327 | 0.00000 |
| O | 2.22605 | -0.18643 | 0.00000 |
| Ketene |  |  |  |
| C | 0.00000 | 0.00000 | 0.10357 |
| C | 0.00000 | 0.00000 | 1.27264 |
| H | 0.00000 | 0.00000 | -1.21741 |
| H | 0.00000 | 0.94052 | -1.74906 |
| O | 0.00000 | -0.94052 | -1.74906 |
| Allene |  |  |  |
| C | 0.00000 | 0.00000 | 1.31430 |
| C | 0.00000 | 0.00000 | -1.31430 |
| H | 0.00000 | 0.92976 | -1.87476 |
| H | -0.92976 | 0.00000 | 1.87476 |
| H | 0.92976 | 0.00000 | 1.87476 |
| C | 0.00000 | 0.00000 | 0.00000 |
| H | 0.00000 | -0.92976 | -1.87476 |
| CO2 |  |  |  |
| C | 0.00000 | 0.00000 | 0.00000 |
| O | 0.00000 | 0.00000 | 1.17001 |
| O | 0.00000 | 0.00000 | -1.17001 |

Table S5. Calculated coordinates at the MP2/6-311++G** level for diketene, ketene, allene and CO_2_

| Atom | *x* | *y* | *z* |
| --- | --- | --- | --- |
| diketene |  |  |  |
| C | 0.05390 | 1.15483 | 0.00000 |
| C | 1.05032 | -0.00505 | 0.00000 |
| H | 0.09180 | 1.77258 | 0.89900 |
| H | 0.09180 | 1.77258 | -0.89900 |
| C | -0.95102 | 0.03187 | 0.00000 |
| C | -2.26762 | -0.15307 | 0.00000 |
| H | -2.92595 | 0.70600 | 0.00000 |
| H | -2.68722 | -1.15149 | 0.00000 |
| O | 0.03851 | -0.97377 | 0.00000 |
| O | 2.22601 | -0.18512 | 0.00000 |
| Ketene |  |  |  |
| C | 0.00000 | 0.00000 | 0.10357 |
| C | 0.00000 | 0.00000 | 1.27182 |
| H | 0.00000 | 0.00000 | -1.21814 |
| H | 0.00000 | 0.94350 | -1.74357 |
| O | 0.00000 | -0.94350 | -1.74357 |
| Allene |  |  |  |
| C | 0.00000 | 0.00000 | -1.31411 |
| C | 0.00000 | 0.00000 | 1.31411 |
| H | 0.00000 | 0.93190 | 1.87109 |
| H | 0.93190 | 0.00000 | -1.87109 |
| H | -0.93190 | 0.00000 | -1.87109 |
| C | 0.00000 | 0.00000 | 0.00000 |
| H | 0.00000 | -0.93190 | 1.87109 |
| CO_2_ |  |  |  |
| C | 0.00000 | 0.00000 | 0.00000 |
| O | 0.00000 | 0.00000 | 1.17001 |
| O | 0.00000 | 0.00000 | -1.17001 |

Table S6. Calculated coordinates at the M06-2X/aug-cc-pVTZ level for diketene, ketene, allene and CO_2_

| Atom | *x* | *y* | *z* |
| --- | --- | --- | --- |
| diketene |  |  |  |
| C | -0.05427 | 1.15048 | 0.00000 |
| C | -1.04383 | -0.00673 | 0.00000 |
| H | -0.08946 | 1.76686 | -0.89436 |
| H | -0.08946 | 1.76686 | 0.89436 |
| C | 0.94967 | 0.03033 | 0.00000 |
| C | 2.24895 | -0.15632 | 0.00000 |
| H | 2.91181 | 0.69443 | 0.00000 |
| H | 2.66431 | -1.15264 | 0.00000 |
| O | -0.04221 | -0.96037 | 0.00000 |
| O | -2.20783 | -0.18739 | 0.00000 |
| Ketene |  |  |  |
| C | 0.00000 | 0.00000 | 0.10322 |
| C | 0.00000 | 0.00000 | 1.25776 |
| H | 0.00000 | 0.00000 | -1.20407 |
| H | 0.00000 | 0.94048 | -1.72848 |
| O | 0.00000 | -0.94048 | -1.72848 |
| Allene |  |  |  |
| C | -1.29924 | 0.00000 | 0.00000 |
| C | 1.29924 | -0.00000 | 0.00000 |
| H | 1.85575 | -0.54508 | 0.75138 |
| H | -1.85577 | 0.75135 | 0.54509 |
| H | -1.85577 | -0.75135 | -0.54506 |
| C | 0.00000 | 0.00000 | -0.00002 |
| H | 1.85582 | 0.54507 | -0.75132 |
| CO_2_ |  |  |  |
| C | 0.00000 | 0.00000 | 0.00000 |
| O | 0.00000 | 0.00000 | 1.15546 |
| O | 0.00000 | 0.00000 | -1.15546 |

Table S7. Energies and corrections for enthalpy (*H*corr), Gibbs Free Energy (*G*corr) and Zero Point Energy (ZPEcorr) at CBS-QB3 and energy at CCSD(T)/CBS and corrections at MP2/cc-pvtz level at different temperatures for all the molecules involved in the calculation of the thermochemical properties of decomposition of diketene.^a^

| Energy and corrections both at CBS-QB3 at 298.15 K | | | |  |
| --- | --- | --- | --- | --- |
| Molecule | Energy | *H*corr | *G*corr | ZPEcorr |
| Diketene | -304.780795 | 0.078433 | 0.043872 | 0.072326 |
| Ketene | -152.372330 | 0.036029 | 0.008647 | 0.031579 |
| Allene | -116.416553 | 0.059597 | 0.032056 | 0.054858 |
| Carbon dioxide | -188.369467 | 0.015286 | -0.008974 | 0.011720 |
| Energy at CCSD(T)/CBS and corrections at MP2/cc-pVTZ at 298.15 K | | | | |
| Diketene | -304.930550 | 0.078806 | 0.044404 | 0.072780 |
| Ketene | -152.444407 | 0.036162 | 0.008685 | 0.031657 |
| Allene | -116.492458 | 0.059902 | 0.031786 | 0.055221 |
| Carbon dioxide | -188.442501 | 0.015128 | -0.009163 | 0.011550 |
| Energy at CCSD(T)/CBS and corrections at MP2/cc-pVTZ at 653 K | | | | |
| Diketene | -304.930550 | 0.095161 | -0.002959 | 0.072780 |
| Ketene | -152.444407 | 0.044713 | -0.027540 | 0.031657 |
| Allene | -116.492458 | 0.070376 | -0.005835 | 0.055221 |
| Carbon dioxide | -188.442501 | 0.021024 | -0.040542 | 0.011550 |
| Energy at CCSD(T)/CBS and corrections at MP2/cc-pVTZ at 823 K | | | | |
| Diketene | -304.930550 | 0.105416 | -0.029711 | 0.072780 |
| Ketene | -152.444407 | 0.049611 | -0.046930 | 0.031657 |
| Allene | -116.492458 | 0.076875 | -0.026440 | 0.055221 |
| Carbon dioxide | -188.442501 | 0.024271 | -0.056956 | 0.011550 |

^a^ All energies are in Hartrees

Table S8: Relative energies of reactants, transition states and products (kJ/mol) at CBS-QB3 and CCSD(T)/CBS level (298.15 K) to produce the kinetic energy plots for the two possible pathways of decomposition for diketene.

| Pathway | Reactants | TS | | Products | |
| --- | --- | --- | --- | --- | --- |
|  |  | CBS-QB3 | CCSD(T)  / CBS | CBS-QB3 | CCSD(T)  / CBS |
| (I) | 0 | 193.7 | 235.8 | 94.8 | 124.5 |
| (II) | 0 | 203.8 | 247.7 | -13.7 | -9.9 |

Table S9. Calculated coordinates at the M06-2X/cc-pVTZ level for TS1 (TS for diketene to ketene).

| Atom | *x* | *y* | *z* |
| --- | --- | --- | --- |
| C | -0.180747 | -0.981686 | 0.555482 |
| C | -1.252720 | -0.246573 | 0.018238 |
| H | -0.158823 | -0.896959 | 1.638167 |
| H | -0.189443 | -2.007819 | 0.188862 |
| C | 0.917266 | 0.180610 | 0.086559 |
| C | 2.009934 | -0.277701 | -0.541118 |
| H | 2.155211 | -1.329217 | -0.719808 |
| H | 2.773024 | 0.425581 | -0.838825 |
| O | 0.403741 | 1.262617 | 0.444975 |
| O | -2.096537 | 0.207448 | -0.567896 |

Table S10. Calculated coordinates at the M06-2X/cc-pVTZ level for TS2 (TS for diketene to allene and CO_2_).

| Atom | *x* | *y* | *z* |
| --- | --- | --- | --- |
| C | -0.059204 | 1.230734 | 0.000006 |
| C | 1.033449 | -0.216537 | 0.000002 |
| H | 0.132680 | 1.771996 | 0.918663 |
| H | 0.132688 | 1.772005 | -0.918643 |
| C | -1.112930 | 0.345621 | -0.000003 |
| C | -2.252305 | -0.258150 | -0.000008 |
| H | -3.137550 | 0.368738 | -0.000006 |
| H | -2.343240 | -1.332246 | -0.000014 |
| O | 0.259596 | -1.184503 | -0.000013 |
| O | 2.185575 | 0.035691 | 0.000015 |

Table S11. Calculated equilibrium constants and rate constants by using KiSThelP version 2021 [1] output files in Java runtime environment and their ratios for diketene decomposition reactions (I) and (II) at different temperatures.^a^

| Temperature / K | Equilibrium constants (*K*) in s^-1^ | | Ratios |
| --- | --- | --- | --- |
| ^#^ | *K*_(I)_ | *K*_(II)_ | *K*_(I)_/*K*_(II)_ |
| 298.15 | 2.62×10^-6^ | 2.80×10^10^ | 9.36×10^-17^ |
| 653.00 | 4.24×10^2^ | 2.82×10^9^ | 1.50×10^-7^ |
| 823.00 | 1.12×10^4^ | 1.83×10^9^ | 6.12×10^-6^ |
|  | Rate constants (*k*) in s^-1^ | |  |
| ^#^ | *k*_(I)_ | *k*_(II)_ | *k*_(I)_/*k*_(II)_ |
| 298.15 | 2.34×10^-21^ | 4.81×10^-23^ | 4.86×10^1^ |
| 653.00 | 2.00×10^-2^ | 5.82×10^-3^ | 3.44 |
| 823.00 | 42.70 | 19.90 | 2.15 |

^a^ *K*_(I)_ and *k*_(I)_ are equilibrium constants and rate constants respectively for reaction (I) at 1 atmospheric pressure. *K*_(II)_ and *k*_(II)_ are those of reaction (II).

^#^ Kinetic calculation from M06-2X/cc-pVTZ level of theory with activated complex parameters are shown in above table S11.

**References**

**1.** Canneaux S., Bohr F., and Hénon E., (2014) KiSThelP: a program to predict thermodynamic properties and rate constants from quantum chemistry results. J Comp Chem 35:82–93
